# Hybridisation as an Ecological Process: Admixed Individuals Contribute Substantially to Canopy Biomass in an Intertidal Macroalgal Hybrid Zone

**DOI:** 10.1002/ece3.74264

**Published:** 2026-08-31

**Authors:** Benedikt Brunner, Jonna Szilveszter, Olga Ortega‐Martínez, Ricardo T. Pereyra, Swantje Enge, Gunilla Toth, Kerstin Johannesson, Lars Gamfeldt

**Affiliations:** ^1^ Department of Marine Sciences, Tjärnö Marine Laboratory University of Gothenburg Strömstad Sweden; ^2^ AstraZeneca Biopharmaceuticals R&D, Discovery Sciences Gothenburg Sweden; ^3^ Department of Clinical Genetics and Genomics, Centre for Medical Genomics Sahlgrenska University Hospital Gothenburg Sweden; ^4^ Gothenburg Global Biodiversity Centre Gothenburg Sweden; ^5^ Centre for Sea and Society Gothenburg Sweden

**Keywords:** canopy structure, environmental gradient, foundation species, *Fucus*, hybrid zone, hybridisation, intertidal, introgression, marine biodiversity

## Abstract

Beyond its relevance for evolutionary biology and genetics, hybridisation is increasingly recognised as an ecological process that can modify community structure, yet the demographic contribution of admixed individuals in natural primary‐producer systems remains poorly resolved. On sheltered shores of the Swedish west coast, the canopy‐forming foundation species *Fucus spiralis* (upper intertidal) and *F. vesiculosus* (mid intertidal) meet across a steep, spatially compressed intertidal gradient. Combining fine‐scale spatial sampling at two sites with high‐resolution genomic data, we asked whether morphologically ambiguous individuals are admixed, how ancestry varies with shore height, and how much admixed individuals contribute to standing canopy biomass across a 30‐cm depth gradient. Genome‐wide structure resolved two parental ancestry groups and a sharply bounded, intermediate centimetre‐scale zone of admixed individuals whose elevated heterozygosity is consistent with recent admixture. They contributed about one‐third of total standing canopy biomass while comprising only about one‐sixth of sampled individuals and appear to be an environmentally constrained demographic component dependent on both parental species rather than a self‐sustaining lineage. Admixed individuals therefore form a substantial part of the canopy in the narrow band where neither parental species performs well. Whether that contribution alters canopy continuity or ecosystem function at the boundary is beyond what these data can establish, and we set it out as a prediction for direct testing rather than as a result. More broadly, hybridisation may act as an ecological process shaping spatial structure in foundation‐species systems, even where its evolutionary outcome is transient.

## Graphical abstract

On sheltered shores of the Swedish west coast, the canopy‐forming seaweeds *Fucus spiralis* and *F. vesiculosus* meet across an intertidal gradient only tens of centimetres wide. Genome‐wide data resolve two parental ancestry groups separated by a sharply bounded zone of admixed individuals whose position on the shore tracks depth. These admixed individuals made up about one‐sixth of those sampled but carried roughly one‐third of the standing canopy biomass at the interface.

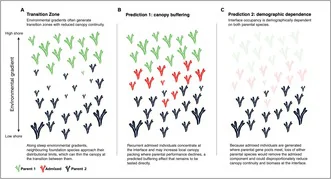

## 1 Introduction

A central principle of modern ecology is that biodiversity drives ecosystem functioning and stability, particularly across larger scales characterised by strong environmental heterogeneity (Gonzalez et al. 2020; Hooper et al. 2005; Loreau et al. 2001). One influential framework explaining this relationship is the spatial insurance hypothesis, which posits that high functioning across the landscape is maintained because species with different abiotic and biotic optima dominate in different environments (Isbell et al. 2018; Loreau et al. 2003). While classical ecology has mostly focused on spatial replacement among distinct species to maintain functioning, intraspecific genetic diversity also plays a critical role in maintaining ecosystem processes (Duffy and Stachowicz 2006; Rieder et al. 2025). Recognising hybridisation as an ecological process thereby links within‐species genetic variation to among‐species turnover in the assembly of communities (Massatti et al. 2025; Porretta and Canestrelli 2023).

Along steep environmental gradients, species can experience sharp physiological thresholds, where small changes in abiotic conditions may push organisms beyond tolerance limits over short distances (Haesen et al. 2023; Osland et al. 2025; Seabra et al. 2011; Zardi et al. 2011). In high‐stress systems, abiotic gradients often place species close to their physiological limits, such that minor environmental changes can result in abrupt declines in performance (Osland et al. 2025). Ecological theory has largely interpreted strong species turnover across environmental gradients as unavoidable consequences of physiological constraints, competitive exclusion, or priority effects (Connell 1961; Farrell 1991; Sousa 1979). Wherever there is turnover in closely related species, it is common to observe hybrids which may have a competitive advantage in the environment where they occur (Porretta and Canestrelli 2023). So far, hybridisation has traditionally been studied primarily from an evolutionary perspective, with emphasis on reproductive isolation and gene flow (R. Abbott et al. 2013; Barton and Hewitt 1985; Ravinet et al. 2017). Recent syntheses argue that hybridisation should also be recognised as an inherently ecological process that is capable of altering species interactions and ecosystem processes, independent of its long‐term evolutionary fate (Porretta and Canestrelli 2023; Whitham et al. 2006). From this perspective, hybrids constitute additional functional elements within communities, potentially having a critical influence on ecosystem functioning wherever they persist.

**FIGURE 1:**
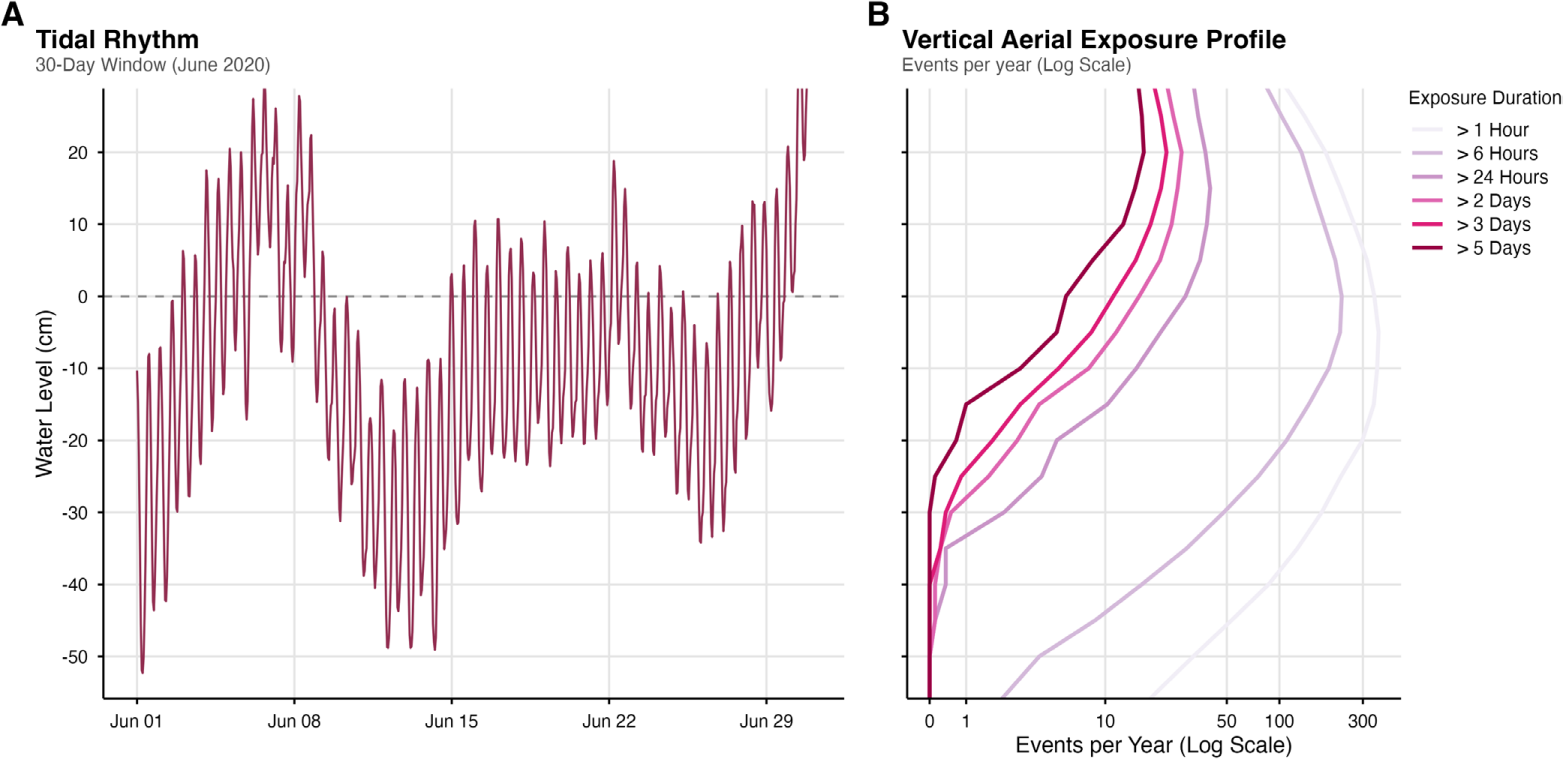
Tidal regime and vertical exposure profile. (A) Hourly sea‐level time series for a representative 30‐day window (June 2020) at the Kungsvik tide gauge; dashed line indicates mean water level (RH2000). (B) Frequency of aerial‐exposure events as a function of shore depth (cm relative to RH2000), calculated from the full 2015–2021 water‐level record for minimum exposure durations (> 1 h, > 6 h, > 24 h, > 2 days, > 3 days, > 5 days; *x*‐axis shown on pseudo‐log scale).

Rocky intertidal shores provide a natural laboratory for testing how parental and hybrid populations coexist because environmental gradients are steep and spatially compressed. On the Swedish west coast, the tide is around 30 cm, but the water level varies up to 1.5 m due to changes in air pressure and onshore and offshore winds (Johannesson 1989). Unpredictable patterns of emersion generate sharp gradients in desiccation stress and temperature variability over vertical distances of only centimetres to decimetres (Figure 1). Canopy‐forming macroalgae act as foundation species in this system, modulating physical stress and structuring diverse associated communities (Cervin et al. 2004; Lubchenco 1980). Fucoid canopies are among the most productive habitats on temperate rocky shores and support diverse understorey and epifaunal communities, so changes in their continuity can have consequences that extend well beyond the canopy itself (Angelini et al. 2011; Thomsen et al. 2024). Among these canopy‐formers, brown fucoid algae of the genus *Fucus* have served as a long‐standing model for studying intertidal zonation, stress physiology, mating system evolution, and hybridisation (Coyer et al. 2011; Hatchett et al. 2022; Thomsen et al. 2024).

On sheltered shores of the Swedish west coast, *Fucus spiralis* and *Fucus vesiculosus* dominate adjacent but largely non‐overlapping vertical zones. *F. spiralis* occupies the upper intertidal, whereas *F. vesiculosus* dominates the lower intertidal (Schrofner‐Brunner et al. 2023). The upper limit of both species is determined by tolerance to abiotic stress, and their lower limits are constrained mainly by competition and herbivory (Billard et al. 2010; Harley 2011; Schonbeck and Norton 1978; Zardi et al. 2011). These species also differ in their mating system: *F. spiralis* is monoecious and predominantly selfing, while *F. vesiculosus* is dioecious and mostly outcrossing (Billard et al. 2010; Engel et al. 2005; Pereyra et al. 2023, 2025). In the intertidal transition zone, individuals occur that cannot be readily assigned to either parental species based on morphology. Hybridisation and gene flow between the two species have been documented throughout their range (Coyer et al. 2011; Pereyra et al. 2025), yet the ecological role of hybrids remains unresolved. Although hybrid zones have been studied intensively from an evolutionary perspective, the contribution of wild hybrids to the structure and functioning of foundation‐species systems, and of marine primary producers in particular, has rarely been examined. More specifically, the contribution of wild admixed individuals to standing canopy biomass in a marine foundation‐species system has, to the best of our knowledge, not previously been quantified. It is thus an open question whether hybridisation is merely a genetic curiosity at boundaries between species, or a structurally important component of a continuous algal canopy.

Zonation among fucoid macroalgae on the Swedish west coast is highly compressed, with dominant species replacing one another over vertical distances of centimetres (Johannesson 1989; Schrofner‐Brunner et al. 2023). In this system, the transition between *F. spiralis* and *F. vesiculosus* occurs across a narrow vertical band where both parental species persist close to their physiological tolerances. Because canopy‐forming fucoids can substantially ameliorate physical stress even at low densities, small changes in canopy identity or continuity may have disproportionate consequences for the microclimate these canopies modulate, and hence also for their associated understorey communities (Harris et al. 2025). The narrow gradient, therefore, provides a rare opportunity to study whether hybridisation merely reflects genetic spillover between stressed populations, or whether it generates a structured demographic unit that contributes substantial standing biomass where the parental species meet.

Here, we study whether hybridisation between *F. spiralis* and *F. vesiculosus* generates a distinct genetic component within the intertidal transition zone that bridges the spatial gap between parental species. Using high‐resolution genomic data combined with centimetre‐scale spatial sampling, we address three related questions:

- What is the genetic ancestry of morphologically ambiguous individuals occurring in the intertidal transition band?
- How does genetic ancestry vary along the vertical intertidal gradient, and is the transition zone characterised by a dominance of admixed genotypes or by a spatial mixture of parental individuals?
- How does accounting for admixed individuals alter the distribution of standing canopy biomass across the intertidal gradient, compared with expectations based on the parental species alone, and what does this imply for canopy continuity at the boundary?

## 2 Material and Methods

### 2.1 Study System and Field Sampling

The study was conducted in the archipelago of the Swedish West Coast (Skagerrak), a system characterised by a compressed intertidal gradient with water‐level variation determined by the combination of a narrow tidal range (amplitude ~35 cm) and the impact of variation in air pressure and wind direction (amplitude > 1.5 m; Figure 1A). Sampling was carried out at two sheltered rocky shore sites near Tjärnö Marine Laboratory: Långholmen (58°53′04.6″ N, 11°07′08.4″ E) and Matkullen (58°53′00.0″ N, 11°07′00.8″ E). The upper and lower boundaries of the canopy formed by *F. spiralis* and *F. vesiculosus* occur over a short vertical range at these shores. We collected thalli across a continuous transect ~30 cm in vertical depth and ~150 cm in length. For each thallus, we recorded its vertical position relative to the mean water level in the Swedish national height system RH2000 (cm; negative values denote depths below the mean water level). Thalli were processed for phenotyping (see below), and a subset was genotyped using reduced‐representation sequencing (see below).

To translate shore depth into biologically meaningful stress regimes, we used water‐level records (2015–2021) from the Swedish Meteorological and Hydrological Institute (SMHI) monitoring station at Kungsvik (58°59′48″ N, 11°07′38″ E). From these records we derived a vertical exposure profile quantifying the expected frequency of air‐exposure events per year across shore depths, including event‐duration classes (Figure 1B). Specifically, for each shore depth we derived time intervals during which water level dropped below that depth (i.e., the organism would be air‐exposed), then summarised counts of exposure events by duration thresholds (> 1 h, > 6 h, > 24 h, > 2 days, > 3 days, > 5 days), expressed as expected events per year.

### 2.2 Phenotypic Measurements

We characterised phenotypic variation along the transect using a combination of continuous traits (functional/morphometric) and discrete traits (morphological features and reproductive state). Trait choice and framing followed established macroalgal trait approaches (Mauffrey et al. 2020).

Discrete morphology and reproductive state were scored visually on fresh material: presence/absence of air bladders, presence/absence of spiral shoots, and reproductive condition (no receptacles; receptacles with only oogonia; only antheridia; or both). Reproductive condition was assigned from receptacle presence and conceptacle content, where applicable. For biomass, each thallus was blotted to remove excess surface water and weighed to obtain wet mass. Because receptacle mass can dominate wet weight and varies with reproductive state, all biomass comparisons used dry mass (see below).

To quantify continuous morphology and area‐based traits (Cappelatti et al. 2019), thalli were photographed on a contrasting background with a scale bar. Images were analysed in ImageJ (Schneider et al. 2012) to extract surface area and perimeter. Subsequently, the whole thalli were dried for 48 h at 60°C and weighed to obtain dry mass. From these measurements we derived STA (specific thallus area, i.e., area per unit dry mass), SAP (surface area‐to‐perimeter ratio), and TDMC (thallus dry matter content, dry mass divided by wet mass).

Phlorotannins were quantified from freeze‐dried tissue using a modified Folin–Ciocalteu assay based on Koivikko et al. (2005). Freeze‐dried samples were homogenised, and approximately 10 mg of tissue was extracted in 1.5 mL 60% acetone for 1 h on a shaking table, followed by centrifugation for 2 min. One millilitre of supernatant was transferred to a new tube, acetone was evaporated in vacuo, and extracts were brought to 10 mL with dH2O and clarified by centrifugation to remove precipitated lipophilic material. For colour development, 0.5 mL diluted extract was mixed with 3.5 mL dH2O, 0.25 mL Folin–Ciocalteu reagent, and 0.75 mL Na_2_CO_3_ (20 g per 100 mL), incubated for 2 h in the dark, and absorbance was measured at 725 nm. Concentrations were calculated from a phloroglucinol standard curve and expressed as percent dry mass. A full step‐by‐step protocol is provided in Methods S1.

Continuous trait variables were centred and scaled (*z*‐scored). We summarised continuous trait space using principal component analysis (PCA) and quantified group differences in multivariate trait space using PERMANOVA, implemented in vegan (Oksanen et al. 2024). Dispersion was checked where relevant to support the interpretation of PERMANOVA results.

### 2.3 DNA Extraction and Genotyping

Genomic DNA was extracted from apical tissue using a DNeasy Plant kit (Qiagen) with a CTAB‐based clean‐up modification where needed (Panova et al. 2016). We generated reduced‐representation libraries using a 2bRAD approach based on BcgI digestion (Wang et al. 2012) with modifications previously applied in *Fucus* (Pereyra et al. 2023). Libraries were sequenced on an Illumina NovaSeq 6000. Technical replication was incorporated by preparing replicate libraries for a subset of individuals; these were used to quantify overall pipeline repeatability and guide filtering decisions. Raw reads were processed with the 2bRAD pipeline: reads were trimmed and deduplicated and retained only when they carried an exact BcgI recognition pattern with no ambiguous bases. High‐quality reads were aligned to the *F. vesiculosus* draft genome assembly ASM1454947v1 (NCBI Assembly; BioProject PRJNA629489) using Bowtie2 (Langmead and Salzberg 2012). The overall mean alignment rate was 67.6% (range 41.6%–82.4%), with no evidence of mapping bias against the non‐reference species (mean 67.0% in *F. spiralis* (*n* = 40) vs. 67.6% in *F. vesiculosus* (*n* = 26)). Variants were called jointly using SAMtools/BCFtools (Danecek et al. 2021), retaining indels during joint calling and excluding them at site filtering when restricting the final dataset to biallelic SNPs; during variant calling, reads were filtered by mapping quality (≥ 20) and base quality (≥ 20). To minimise biases from low‐confidence genotypes, we first applied genotype‐level masking: genotypes with low read depth or low genotype quality were set to missing (DP < 3 or GQ < 20). After genotype masking, we applied site‐level filters to the resulting SNP set to remove loci with high missingness and very rare alleles, and to control for depth outliers: loci with unusually high total depth were removed using a fixed site‐level depth filter (INFO/DP > 3850), together with F_MISSING > 0.20 and MAF < 0.01. No LD pruning was applied to the fixed‐reference SNP dataset used for semi‐diagnostic marker discovery and HI estimation; LD pruning was applied only for the independent genome‐wide Q analysis described below. After genotype‐level masking and site‐level filtering, the final fixed‐reference dataset retained 5042 biallelic SNPs across 80 non‐replicate individuals. Across these 80 individuals, mean called‐genotype depth was 16.5× (range 10.7–44.1×), and the mean per‐individual call rate was 97.4% (range 84.3%–100%). For ancestry estimation with sNMF, LD pruning retained 4155 SNPs (*K* = 2 analysis).

### 2.4 Data Analysis

For genome‐wide structure, we used both PCA and model‐based ancestry coefficients (*Q*). PCA was computed from the filtered genome‐wide SNP set (excluding technical replicates). We estimated *Q* at *K* = 2 using sNMF in LEA (Frichot et al. 2014) on an LD‐pruned SNP set generated with SNPRelate (Zheng et al. 2012).

Because the two taxa form a gradient with limited fixed divergence, we defined fixed ecological reference groups using extreme shore‐depth positions rather than assuming discrete populations. High‐shore references were defined as individuals at the upper end of the transect consistent with *F. spiralis* morphology, and low‐shore references as individuals at the lower end consistent with *F. vesiculosus* morphology. The two ecological reference groups (high shore: depth > −6 cm, *n* = 13; low shore: depth < −20 cm, *n* = 12) were used only to compute per‐locus allele‐frequency differences (ΔAF) and orient allele coding; the strict hybrid index (HI) was then calculated as the raw mean low‐shore allele dosage across retained loci (ΔAF ≥ 0.8) for each individual.

To estimate hybrid ancestry in a low‐divergence system, we used semi‐diagnostic loci defined by ΔAF between reference groups, rather than requiring fixed differences (Salces‐Castellano, Stankowski, et al. 2021). For each SNP, we calculated ΔAF between low‐shore and high‐shore references and retained loci exceeding a threshold. With fixed ecological references, marker counts were 30 loci at ΔAF ≥ 0.5 and 4 loci at ΔAF ≥ 0.8 (Figure S3).

We quantified ancestry using the hybrid index (HI) (Buerkle 2005; Salces‐Castellano, Andújar, et al. 2021). For each semi‐diagnostic SNP, we oriented allele coding so that the low‐shore allele was more frequent in the low‐shore reference set, then coded genotypes as 0/1/2 copies of this allele. For the main analysis, we used the strict set (ΔAF ≥ 0.8; 4 loci): strict HI was the mean low‐shore allele dosage divided by two.

Individuals were assigned to three groups from the strict hybrid index: < 0.05 (parental *F. spiralis* ; *n* = 40), 0.05–0.95 (admixed; *n* = 14), and > 0.95 (parental *F. vesiculosus* ; *n* = 26). These three groups were used in the downstream trait and biomass analyses.

For a supplementary robustness check, we re‐derived semi‐diagnostic markers from alternative reference groups defined by genome‐wide *Q* extremes (15 lowest‐*Q* and 15 highest‐*Q* individuals; *K* = 2), which yielded 29 loci at ΔAF ≥ 0.5. Figure S4 compares the strict hybrid index (fixed‐reference, ΔAF ≥ 0.8; 4 loci) with the raw hybrid index from this *Q*‐reference set, confirming that the ancestry estimates are not an artefact of the small marker panel.

We evaluated how ancestry varies across the environmental gradient using shore depth and the strict hybrid index, summarised with a Spearman rank correlation and visualised with non‐parametric smoothing (LOESS) and 95% bands. All analyses were conducted in R 4.5.2 using a fully scripted workflow covering phenotypic processing, bioinformatics summaries, ancestry and heterozygosity estimation, and statistical analyses.

## 3 Results

Our analysis of population structure identified two main ancestral groups (*K* = 2), which correspond to the parental species *F. spiralis* and *F. vesiculosus* . As expected from their different mating systems, these parental clusters showed highly divergent genetic diversity profiles. Between these extremes we found a set of admixed individuals with intermediate ancestry. The two sites did not separate in genome‐wide structure and were therefore pooled for further analysis (Figure S2).

**FIGURE 2:**
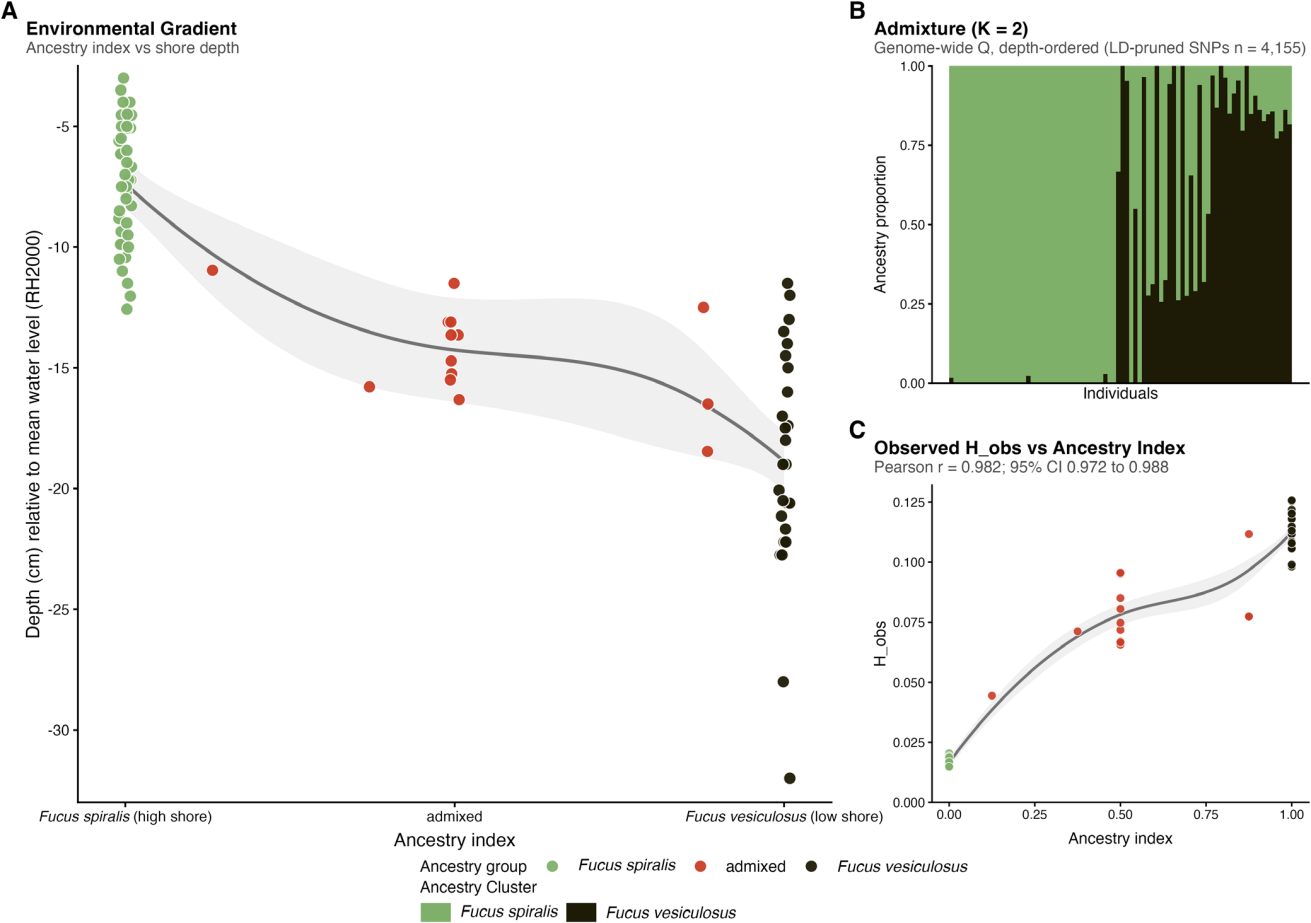
Environmental gradient and genomic ancestry across individuals. (A) Shore depth (cm relative to RH2000) versus the strict hybrid index (strict HI) estimated from semi‐diagnostic loci (ΔAF ≥ 0.8; 4 loci), with LOESS smoother and 95% confidence band. (B) Genome‐wide ancestry coefficients (*Q*, *K* = 2) inferred independently with sNMF from LD‐pruned SNPs (*n* = 4155); individuals are ordered by shore depth, and bars show the two ancestry components (*spiralis*‐like and *vesiculosus*‐like). (C) Genome‐wide observed heterozygosity (H_obs; 5042 SNPs) versus the strict hybrid index, with LOESS smoother and 95% confidence band. HI, hybrid index; H_obs, observed heterozygosity; *Q*, ancestry coefficient; ΔAF, absolute allele‐frequency difference.

Shore depth and genomic ancestry were tightly linked across the full set of non‐replicate genotyped individuals (*n* = 80; Figure 2A–C). The strict hybrid index (HI; ΔAF ≥ 0.8, 4 loci) increased from shallow to deep shore positions (Spearman rho = −0.870, *p* < 0.001; depth is negative below mean water level; Figure 2A). Using the three‐group scheme, depth distributions were ordered along the shore: parental *F. spiralis* mean −7.4 cm (range −12.6 to −3.0), admixed mean −14.4 cm (range −18.5 to −11.0), and parental *F. vesiculosus* mean −18.9 cm (range −32.0 to −11.5), consistent with a continuous gradient of admixture tightly constrained within the compressed shore gradient (Figure 2A).

Divergence between the two taxa was incomplete at the marker level. Across 5042 filtered SNPs, only 30 loci met semi‐diagnostic differentiation (ΔAF ≥ 0.5), and only 4 loci met the strict threshold (ΔAF ≥ 0.8; fixed in the ecological references; Figure S3). Genome‐wide observed heterozygosity tracked the strict hybrid index closely (Pearson *r* = 0.982, 95% CI 0.972–0.988, *p* < 0.001; Figure 2C), with group means of 0.0165 in parental *F. spiralis* (range 0.0147–0.0204), 0.0775 in admixed individuals (0.0444–0.1117), and 0.1129 in parental *F. vesiculosus* (0.0982–0.1257). The intermediate heterozygosity of admixed individuals is consistent with mixed ancestry in the context of the contrasting parental mating systems; because the strict panel contains only 4 loci, we do not assign generation classes. These relationships were reproduced using genome‐wide ancestry (*Q*) in place of the four‐locus index (Figure S5), confirming they are not artefacts of the small marker panel.

**FIGURE 3:**
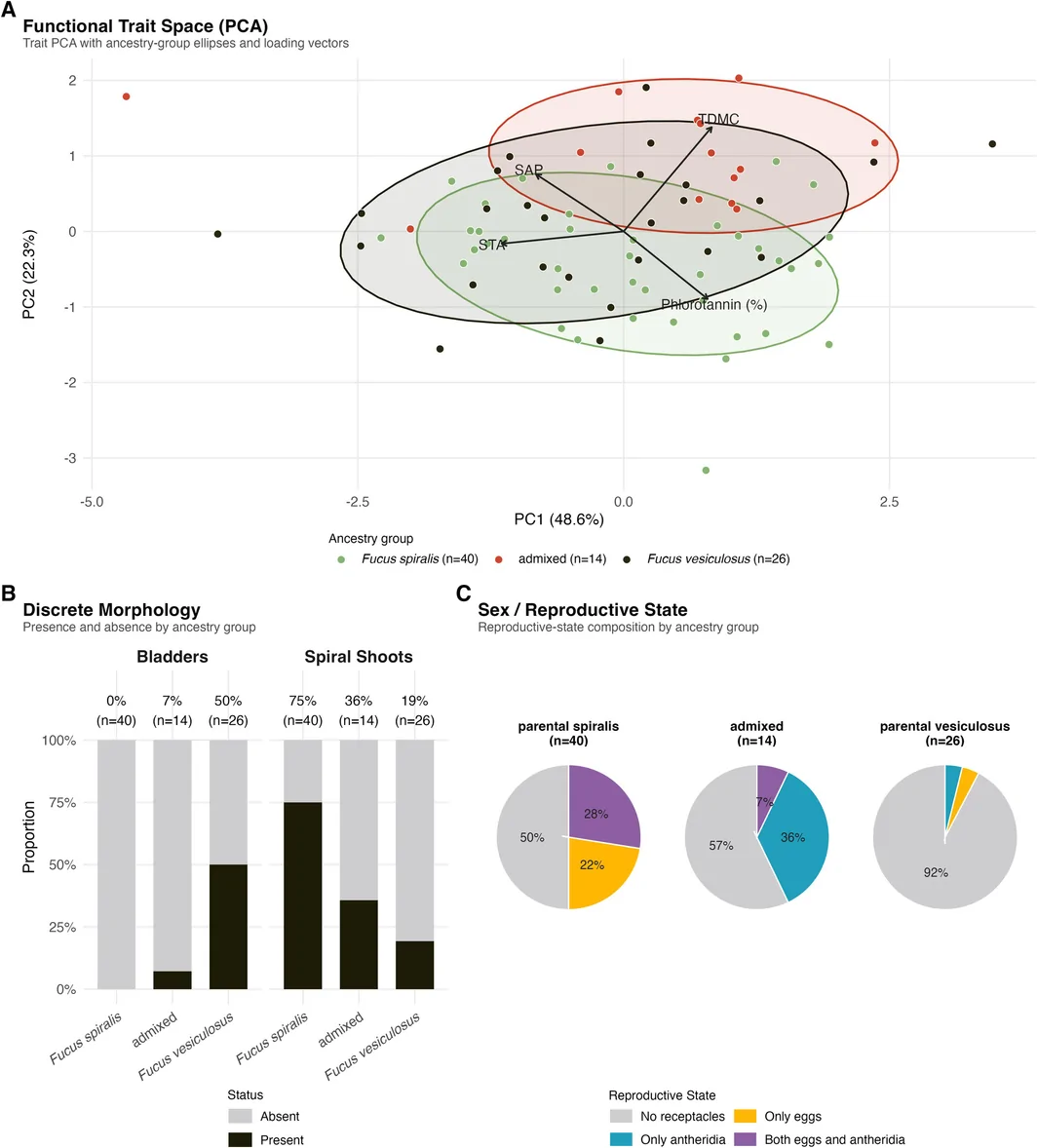
Functional and reproductive trait structure across ancestry groups. (A) PCA of centred and scaled continuous traits (phlorotannin % dry mass, STA, SAP, TDMC) with 80% group ellipses and trait loading vectors. (B) Discrete morphology: Proportion of individuals with gas vesicles (“bladders”) and spiral shoots across the three ancestry groups (parental *Fucus spiralis* , admixed, parental *F. vesiculosus* ); sample sizes shown above bars. (C) Reproductive‐state composition for parental *F. spiralis* , admixed individuals, and parental *F. vesiculosus* (pie charts; categories: No receptacles, eggs only, antheridia only, both). PCA, principal component analysis; SAP, surface area‐to‐perimeter ratio; STA, specific thallus area (cm^2^ g^−1^); TDMC, thallus dry matter content.

Phenotypic structure supported differentiation between the parental groups and admixed individuals (Figure 3A,B). In continuous trait space (phlorotannin, STA, SAP, TDMC), PCA axes explained 48.6% (PC1) and 22.3% (PC2) of the variance. A global multivariate effect of group on trait composition was present (PERMANOVA *R* ^2^ = 0.089, *F* = 3.77, *p* = 0.002), with no evidence that it was driven by unequal dispersion (PERMDISP *F* = 0.82, *p* = 0.448). After Benjamini–Hochberg adjustment, the clearest pairwise contrast was between parental *F. spiralis* and admixed individuals (*p* = 0.002), whereas the parental *F. spiralis* versus parental *F. vesiculosus* (*p* = 0.065) and admixed versus parental *F. vesiculosus* (*p* = 0.080) contrasts were not significant.

Univariate trait summaries varied across groups, though ranges overlapped substantially and the clearest separation was multivariate (Figure 3A). Phlorotannin was highest in *F. spiralis* (mean 9.44%, range 6.92–12.58), lower in admixed individuals (8.50%, 6.23–11.74), and intermediate in *F. vesiculosus* (8.97%, 4.81–12.52). STA was lowest in admixed individuals (41.5, 28.8–70.0), intermediate in *F. spiralis* (45.5, 25.2–70.1), and highest in *F. vesiculosus* (49.2, 25.3–83.9). SAP was highest in admixed individuals (0.308, 0.178–0.682), lower in *F. vesiculosus* (0.285, 0.188–0.371), and lowest in *F. spiralis* (0.243, 0.132–0.486). TDMC was also highest in admixed individuals (0.292, 0.228–0.332), intermediate in *F. vesiculosus* (0.265, 0.204–0.356), and lowest in *F. spiralis* (0.254, 0.188–0.310). Discrete morphology showed a mixed/intermediate pattern: bladders were absent in *F. spiralis* (0/40) and rare in admixed individuals (1/14, 7.1%) but present in *F. vesiculosus* (13/26, 50.0%), while spiral shoots were frequent in *F. spiralis* (30/40, 75.0%), intermediate in admixed individuals (5/14, 35.7%), and least frequent in *F. vesiculosus* (5/26, 19.2%) (Figure 3A,B).

Reproductive‐state distributions highlighted a notable but low‐n sex pattern in admixed individuals (Figure 3C). Among the 14 admixed individuals, 8 had no receptacles, 5 had antheridia only, and 1 had both eggs and antheridia; none bore eggs only. In *F. spiralis* , when receptacles could be scored, both eggs and antheridia were common (11/20), while 9/20 were scored as eggs‐only; these eggs‐only records are compatible with reduced detectability of antheridia late in the reproductive season. The largely male‐biased reproductive pattern in admixed individuals should therefore be treated as provisional until evaluated in larger samples across seasons (Figure 3C).

**FIGURE 4:**
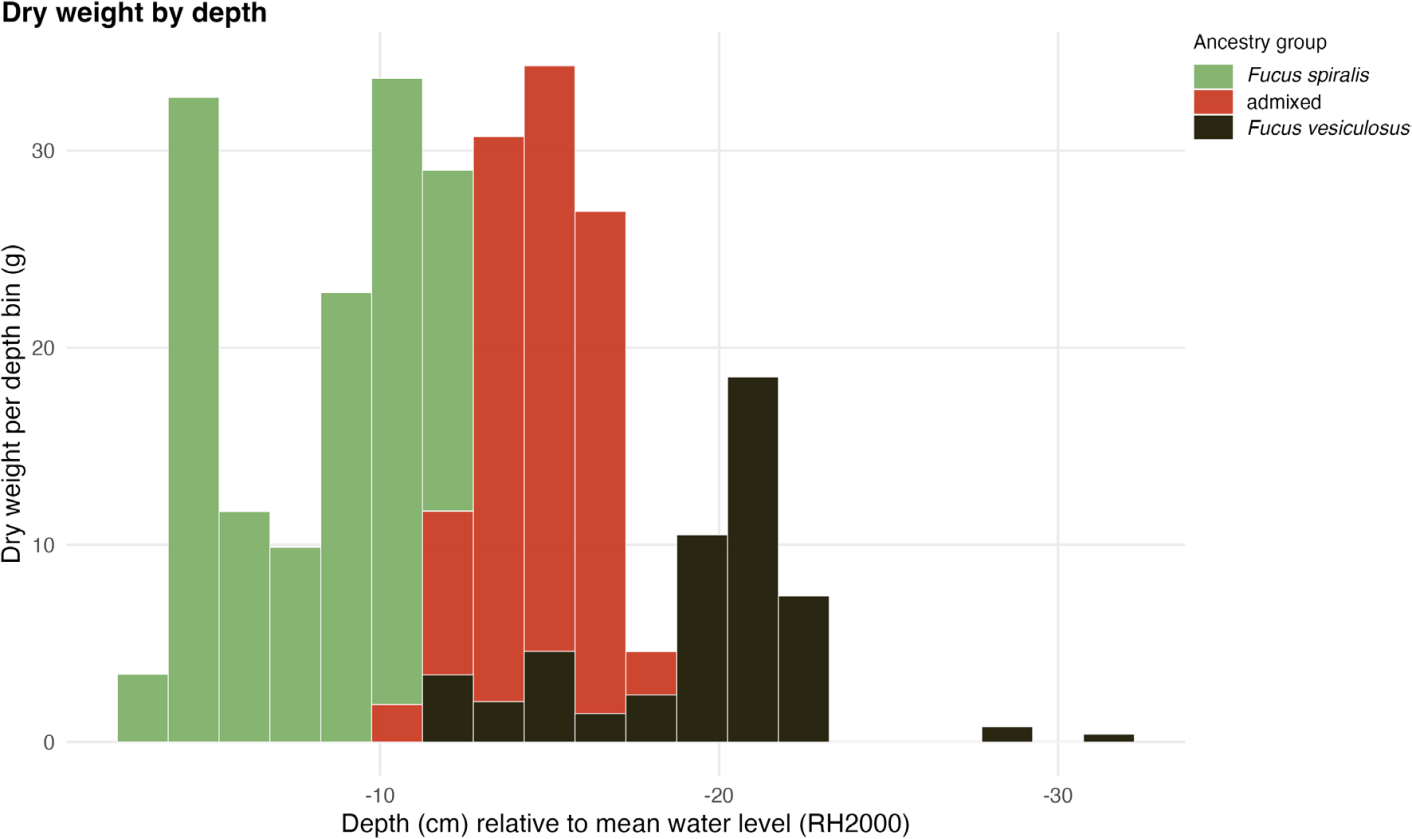
Depth distribution of dry biomass across ancestry groups. Biomass‐weighted histogram of dry biomass (g) versus shore depth (cm relative to RH2000) for all individuals (*n* = 80). Colours indicate the three ancestry groups (parental *Fucus spiralis* , admixed, parental *F. vesiculosus* ); *x*‐axis is reversed.

Admixed individuals contributed substantially to standing biomass (Figure 4). Of the total dry biomass (277 g), admixed individuals (*n* = 14) accounted for 96.2 g, or 34.7%, while making up only 14 of the 80 sampled individuals (17.5%).

Technical replicate QC supported overall genotype reliability (16 replicate‐original pairs: median concordance 0.999, mean 0.981), with two low‐concordance pairs (minimum 0.833) retained in the supplementary QC reporting (Figure S1).

## 4 Discussion

Fine‐scale sampling of genomic ancestry at centimetre resolution along a steep vertical shore gradient revealed a sharply bounded hybrid zone between *F. spiralis* and *F. vesiculosus* . Admixed individuals were concentrated within a narrow mid‐shore band, and genomic ancestry turned over rapidly across a depth interval of approximately 10–12 cm. Although the depth ranges of the three groups overlapped (Figure 2A), their central positions were strongly ordered along the shore, indicating that the rapid ancestry turnover reflects a real shift in the centre of each group's distribution rather than fully exclusive zonation. This abrupt transition coincides with a steep abiotic gradient, in which small changes in shore height translate into large differences in emersion duration and associated desiccation and thermal stress (Figure 1B). *Fucus spiralis* occupied the upper intertidal, *F. vesiculosus* dominated the lower intertidal, and admixture was rare outside the transition zone, consistent with strong spatial structuring across a highly compressed environmental boundary. This is consistent with the general fucoid pattern that upper shore limits are set primarily by abiotic stress and lower limits by biotic interactions such as competition (Harley 2011; Schonbeck and Norton 1978). Within this band, *F. spiralis* approaches its lower limit and *F. vesiculosus* its upper limit.

The genetic composition of the hybrid zone suggests that admixed individuals are not forming an independent, self‐sustaining entity. We do not assign precise generation classes, because the genome‐wide data and the elevated heterozygosity of admixed individuals are sufficient to establish recent admixture without resolving F1s from later‐generation hybrids or backcrosses. Genome‐wide observed heterozygosity in admixed individuals was intermediate between the parental groups (Figure 2C), consistent with a substantial component of recent admixture rather than a long‐established recombinant swarm. A predominance of recent admixture is expected where selection changes sharply with the environment, or where admixed individuals experience intrinsic incompatibilities and reduced fertility (Barton and Hewitt 1985; Milne et al. 2003). Our reproductive observations are broadly consistent with reduced fertility but should be treated cautiously given small sample sizes: most admixed individuals were non‐reproductive or bore only antheridia, and only one bore both eggs and antheridia (Figure 3C). The recovery of intermediate ancestry across the zone indicates that admixed individuals are not uniformly sterile and can contribute to backcrossing. Given limited sample sizes and seasonal constraints, the reproductive pattern should be re‐evaluated across the reproductive season.

Observed heterozygosity patterns are also consistent with recent admixture in the context of mating system asymmetry. The predominantly selfing, hermaphroditic *F. spiralis* displayed markedly low heterozygosity, whereas the dioecious *F. vesiculosus* showed substantially higher heterozygosity (Billard et al. 2010; Engel et al. 2005). Importantly, these absolute differences reflect true biological distinctions rather than mapping artefacts, because read alignment rates to the *F. vesiculosus* reference genome were equivalent for both parental species. Admixed individuals displayed elevated heterozygosity compatible with transient heterosis, offering a demographic explanation for why they can repeatedly establish within the interface even when introgression remains limited (Barton and Hewitt 1985; Hatchett et al. 2022).

Trait patterns further sharpen the interpretation of why hybrids remain confined to the interface. The parental species overlapped partially in multivariate space for continuous traits, and *F. spiralis* was associated with traits linked to desiccation tolerance, such as higher phlorotannin content (Henry and Van Alstyne 2004; Meichssner et al. 2023). In the multivariate trait space, admixed individuals overlapped most with *F. vesiculosus* . Discrete morphological traits reinforce this constraint: gas vesicles, common in low‐shore *F. vesiculosus* , were absent in *F. spiralis* and rare among admixed individuals (1 of 14), while spiral shoots were common in *F. spiralis* , less frequent in admixed individuals, and least frequent in *F. vesiculosus* . These patterns point to an interface niche where hybrids are poorly suited to either extreme: the lack of gas vesicles may constrain performance at lower shore levels, while incomplete expression of high‐shore morphology may limit persistence at higher shore levels. A key limitation is that our trait set likely captures structural proxies more effectively than the metabolic and physiological mechanisms that could be decisive for fitness (Cappelatti et al. 2019; Rustas et al. 2026).

These results address long‐standing uncertainty about the nature of “intermediate” plants at *Fucus* zone boundaries. Historically, ambiguous individuals were often interpreted as environmentally induced ecads or transient overlaps because diagnostic traits can be plastic and stress‐modulated (Coyer et al. 2006; Schonbeck and Norton 1978; Zardi et al. 2011). Since *F. vesiculosus* and *F. spiralis* can both show substantial morphological variation across environmental conditions, morphology alone is likely insufficient to distinguish all boundary individuals reliably; genomic data are therefore essential for separating parental and hybrid ancestry in this system. By resolving genomic ancestry directly, we show that many intermediate thalli at the boundary represent genuinely admixed individuals, not merely plastic parental morphotypes. This supports the view that hybridisation is a recurring, spatially structured component of the *Fucus* zone (Coyer et al. 2011; Hatchett et al. 2022).

The *Fucus* system fits a broader class of tension zones in which steep environmental gradients generate spatially confined hybrid populations that remain demographically dependent on continuous gene flow from parental taxa. In plants, hybrid zones along sharp ecological transitions can be narrow and dominated by hybrids when selection is strong (Abbott 2017; Abbott and Brennan 2014; Kimball et al. 2008; Milne et al. 2003). In animals, replicated hybrid zones in *Xiphophorus* swordtails correspond to elevational and thermal clines and repeatedly restrict hybrids to intermediate environments (Culumber et al. 2011). These systems differ in reproductive biology and selective agents, yet converge on the same emergent pattern: strong, spatially structured environmental heterogeneity can stabilise hybrids as geographically restricted, recurrent products of gene flow rather than expanding lineages. This framing is important for interpretation in *Fucus*: our results speak to the ecological consequences of hybridisation without implying hybrid speciation such as in *Spartina*, where hybridisation (often coupled with polyploidy) has been associated with rapid spread, increased dominance, and displacement of native vegetation by expansion of hybrid lineages (Ainouche and Gray 2016; Ayres et al. 2008; Castillo et al. 2010). By contrast, the *Fucus* hybrid zone described here remains tightly constrained to a narrow band and does not form an advancing front; the most plausible ecosystem‐relevant pathway is therefore local niche completion rather than invasion or broad spatial expansion.

Functional analogies also extend beyond plants to include “frozen” hybrid entities and their ecological roles. Hybrid‐derived, parthenogenetic vertebrates offer a conceptually similar, though mechanistically distinct, route to ecological relevance: hybrid origin can generate entities that occupy environmental space differently from their sexual relatives, even when subsequent evolutionary dynamics are constrained. In parthenogenetic rock lizards, hybrid origin produces lineages that are effectively “frozen” genetically relative to sexual hybrid zones, yet these lineages can still differ in habitat use and thereby reshape spatial patterns of occupancy (Neaves and Baumann 2011; Petrosyan et al. 2020). The analogy to *Fucus* is functional rather than mechanistic, underscoring that hybrid origin can have ecological relevance even without hybrid speciation and even when the hybrid entity is maintained by different demographic processes. Although partial clonality has been reported for *F. vesiculosus* , particularly in the northern Baltic (Pereyra et al. 2023), we did not find evidence for any of our samples being of clonal origin. This perspective supports arguments that hybridisation should also be considered an ecological process with potential community and ecosystem consequences, independent of whether it results in the formation of hybrid species (Porretta and Canestrelli 2023; Whitham et al. 2006).

### 4.1 Contribution of Admixed Individuals to Canopy Structure

Distinct canopy entities, including a high‐biomass admixed component, together span the steep environmental gradient. Whether this arrangement sustains canopy occupancy across the gradient, in the sense of the spatial insurance hypothesis (Loreau et al. 2003), is a question our data raise rather than answer. Because fucoid algae function as foundation species that buffer physical stress, even modest changes in canopy continuity at the abiotic boundary can disproportionately influence understorey microclimate and community persistence (Angelini et al. 2011; Harris et al. 2025; Thomsen et al. 2024). What our results do establish is that the contribution of admixed individuals is not marginal: they contributed about one‐third of the standing dry biomass within the surveyed interface.

**FIGURE 5:**
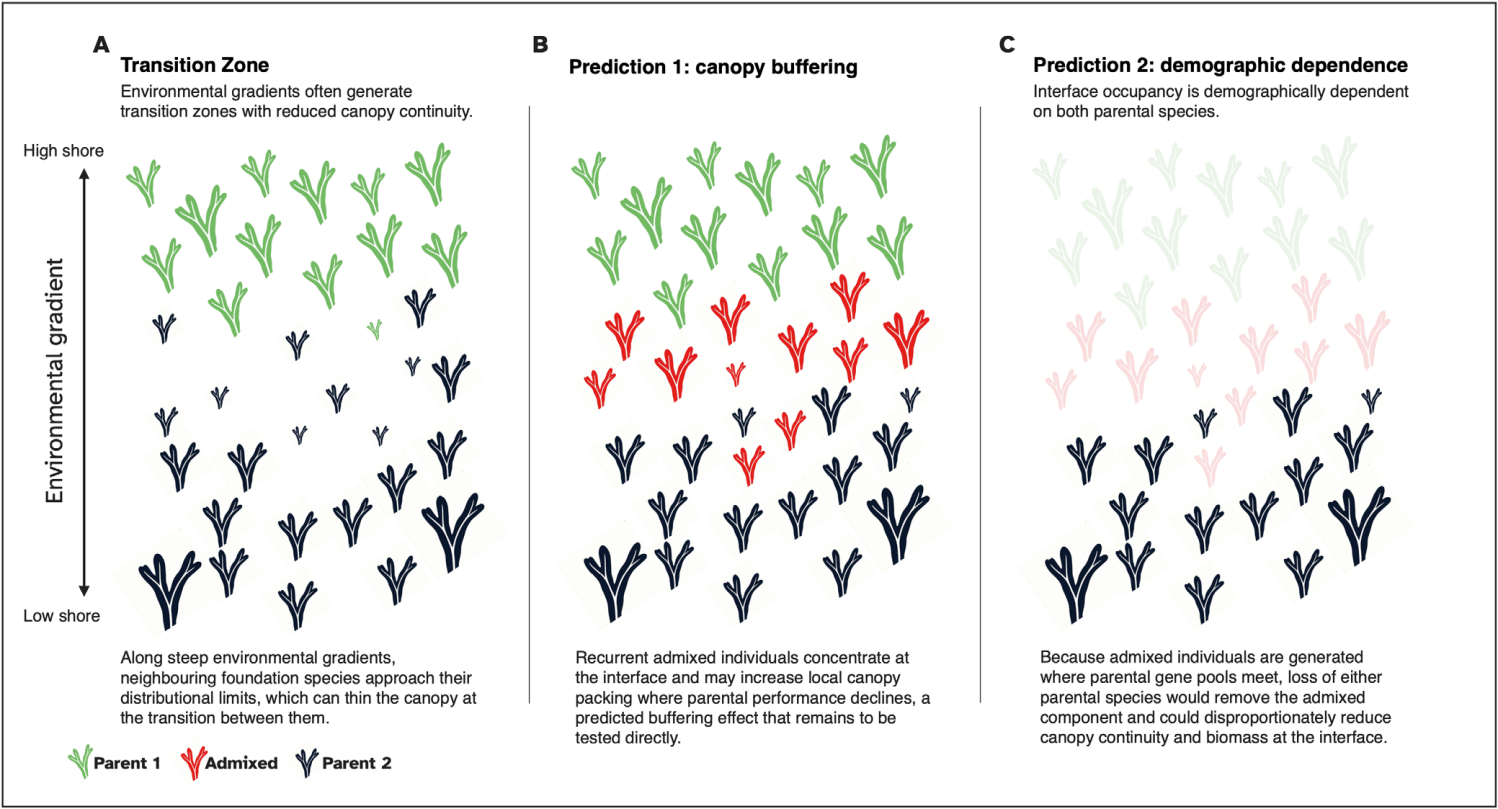
Conceptual model of hybrid‐mediated canopy structure along a steep environmental gradient. (A) Sharp gradients can segregate foundation species and create narrow transition zones between them. (B) Where hybridisation occurs, recurrent hybrids may contribute to canopy continuity at this boundary while remaining spatially restricted and demographically dependent on parental populations. (C) This dependency implies that loss of either parental species could disproportionately affect canopy structure at the interface and highlights how hybridisation can interact with facilitation in heterogeneous environments.

Because hybrids form repeatedly where the two species meet, they constituted a structural component in the band between the physiological limits of the parental species. At the same time, biomass is not identical to canopy cover or packing, and desiccation buffering is expected to be density‐dependent (Harris et al. 2025); the biomass signal therefore motivates but does not, by itself, demonstrate stronger microclimate buffering where hybrids are abundant. The system nevertheless illustrates an interaction between classical species turnover and intraspecific diversity (Duffy and Stachowicz 2006; Porretta and Canestrelli 2023): three distinct canopy entities, two parental species plus a high‐biomass transient hybrid component, partition an extreme and spatially compressed gradient between them (Figure 5). Because admixed individuals do not represent independent lineages but rather a recurring demographic component generated where parental gene pools meet, any functional contribution depends on the continued local presence of both parental taxa and on the boundary conditions that bring them into contact (Figure 5). These possibilities remain untested here. They yield two predictions: first, sites with greater hybrid contribution should show stronger microclimate buffering and different understorey or consumer responses across the interface; second, admixed contributions to canopy cover (as distinct from biomass) should vary across sites and seasons in ways that track the steepness and position of the abiotic transition.

### 4.2 Scope and Limitations

As this is an observational study, the role of selection is inferred primarily from tight genotype–shore coupling rather than from direct measurements of fitness components. Recruitment dynamics and early life‐stage survival were not quantified, although these processes can strongly influence hybrid‐zone maintenance in systems with pronounced gradients and strong environmental filtering. Reciprocal transplants, common‐garden assays, and life‐stage‐specific stress experiments would enable direct estimation of genotype‐by‐environment fitness landscapes and test whether the hybrid zone is maintained primarily by exogenous selection, intrinsic incompatibilities, or their interaction (Kimball et al. 2008; Zardi et al. 2011). We also note that the abiotic gradient itself was characterised from a tidal‐exposure model (Figure 1B) rather than from in situ microclimate measurements along the transects; direct logging of temperature, desiccation, and emersion at the scale of the transition zone would more firmly establish the environmental cause of the observed turnover. Our inferences rest on transects at two sheltered sites on a single coastline. This design is well suited to resolving fine‐scale spatial structure within a hybrid zone, which was our aim, but it cannot establish that hybrid zones in *Fucus* act as canopy bridges generally. To our knowledge, the contribution of admixed individuals to canopy structure in a foundation‐species system has not been examined before, and we therefore present the functional interpretation as a hypothesis for future testing. Replication across additional shores, wave‐exposure regimes, and seasons, paired with direct measures of canopy cover and microclimate and ideally with removal experiments, is needed to test the generality of the pattern and the functional predictions above. We deliberately refrain from assigning precise generation classes; broader marker panels and temporal replication would be required to characterise the generational composition of the zone.

## 5 Conclusion

Within a steep and spatially compressed intertidal gradient, hybridisation between *F. spiralis* and *F. vesiculosus* produces a sharply bounded, centimetre‐scale hybrid zone occupied by admixed individuals with elevated heterozygosity, consistent with recent admixture. These admixed individuals do not appear to form an independent evolutionary lineage; rather, they constitute an environmentally constrained demographic component maintained at the interface where parental performance likely declines. By occupying this transitional band, admixed individuals form a substantial part of the canopy where neither parental species performs well, an ecological consequence that arises even when the evolutionary fate of hybrids remains transient. Whether their presence alters canopy function at the boundary remains to be tested. More broadly, these findings support the perspective that hybridisation can function as an ecological process (Porretta and Canestrelli 2023) that alters spatial structure in communities, complementing classical species‐turnover mechanisms (Loreau et al. 2003) across heterogeneous landscapes.

## Author Contributions

**Benedikt Brunner:** conceptualization (lead), data curation (lead), formal analysis (lead), funding acquisition (equal), investigation (lead), methodology (equal), project administration (equal), resources (lead), software (lead), supervision (lead), validation (lead), visualization (lead), writing – original draft (lead), writing – review and editing (lead). **Jonna Szilveszter:** conceptualization (lead), data curation (lead), formal analysis (lead), funding acquisition (equal), investigation (equal), methodology (lead), project administration (equal), resources (equal), visualization (equal), writing – original draft (lead), writing – review and editing (equal). **Olga Ortega‐Martínez:** investigation (equal), methodology (equal), resources (lead), supervision (equal). **Ricardo T. Pereyra:** conceptualization (equal), formal analysis (equal), investigation (equal), methodology (equal), resources (equal), software (equal), supervision (equal), writing – review and editing (equal). **Swantje Enge:** methodology (equal), resources (equal), supervision (equal), writing – review and editing (equal). **Gunilla Toth:** conceptualization (supporting), project administration (equal), supervision (equal), writing – review and editing (equal). **Kerstin Johannesson:** conceptualization (equal), investigation (equal), methodology (equal), supervision (equal), writing – original draft (equal), writing – review and editing (equal). **Lars Gamfeldt:** conceptualization (lead), funding acquisition (lead), investigation (equal), project administration (equal), resources (equal), supervision (lead), writing – original draft (equal), writing – review and editing (equal).

## Funding

This work was supported by Vetenskapsrådet (2020‐03521).

## Conflicts of Interest

The authors declare no conflicts of interest.

## Supporting information


**Figure S1:** Technical replicate concordance (QC). Exact genotype concordance between technical replicate and original libraries across filtered SNPs (*n* = 16 replicate pairs). Bars show per‐pair concordance; dashed line marks 0.98. QC, quality control; SNP, single nucleotide polymorphism.
**Figure S2:** Genome‐wide genetic structure (all filtered SNPs). PCA of the main filtered SNP dataset (5042 SNPs; *n* = 80). Points are coloured by shore depth (cm relative to RH2000) and shaped by sampling site (A/B). PCA, principal component analysis; PC, principal component; SNP, single nucleotide polymorphism; RH2000, Swedish height datum.
**Figure S3:** Ancestry‐informative loci across ΔAF thresholds. Number of loci retained as ancestry‐informative as a function of ΔAF threshold (absolute allele‐frequency difference between high‐shore and low‐shore anchor sets). ΔAF, absolute allele‐frequency difference.
**Figure S4:** Robustness of hybrid index estimates to marker set and reference definition. Comparison of strict raw hybrid index (strict HI; fixed ecological references, ΔAF ≥ 0.8; 4 loci) versus semi‐diagnostic raw hybrid index from the Q‐defined 15/15 reference workflow (ΔAF ≥ 0.5; 29 loci). Each point is one individual; the dashed line indicates 1:1 agreement, and the solid line shows the linear fit. Colours indicate the three ancestry groups (parental *Fucus spiralis* , admixed, parental *F. vesiculosus* ).
**Figure S5:** Ancestry‐depth and heterozygosity relationships using genome‐wide ancestry (Q). As Figure 2A,C, but with the genome‐wide *K* = 2 *Fucus vesiculosus* ‐like ancestry proportion (Q; 4155 LD‐pruned SNPs) on the x‐axis in place of the 4‐locus ancestry index. A. Genome‐wide Q versus shore depth (Spearman rho = −0.781, *p* < 0.001; LOESS +95% band). B. Genome‐wide observed heterozygosity (H_obs; 5042 SNPs) versus Q (Pearson *r* = 0.974, 95% CI 0.960–0.983). Points coloured by the three ancestry groups. The relationships match those based on the 4‐locus index, confirming they are not artefacts of the small marker panel.
**Methods S1**. Phlorotannin analysis.

## Data Availability

Processed data, metadata and all analysis scripts are deposited in Dryad (datadryad.org/dataset/doi:10.5061/dryad.69p8cz9k9).
