# Supplementary material for: Hybridisation as an Ecological Process: Admixed Individuals Contribute Substantially to Canopy Biomass in an Intertidal Macroalgal Hybrid Zone
Source: Ecol Evol. 2026 Aug 31;16(9):e74264. doi: 10.1002/ece3.74264 (PMC13527866; doi:10.1002/ece3.74264)
Supplement: Supplementary file 1 [file ECE3-16-e74264-s001.docx]

Hybridisation as an ecological process: admixed individuals contribute substantially to canopy biomass in an intertidal macroalgal hybrid zone

Supplementary Data

**Supplementary Figure S1. Technical replicate concordance (QC).** Exact genotype concordance between technical replicate and original libraries across filtered SNPs (n = 16 replicate pairs). Bars show per-pair concordance; dashed line marks 0.98. *Abbreviations:* QC, quality control; SNP, single nucleotide polymorphism.

**Supplementary Figure S2. Genome-wide genetic structure (all filtered SNPs).** PCA of the main filtered SNP dataset (5,042 SNPs; n = 80). Points are coloured by shore depth (cm relative to RH2000) and shaped by sampling site (A/B). *Abbreviations:* PCA, principal component analysis; PC, principal component; SNP, single nucleotide polymorphism; RH2000, Swedish height datum.

**Supplementary Figure S3.** Ancestry-informative loci across ΔAF thresholds. Number of loci retained as ancestry-informative as a function of ΔAF threshold (absolute allele-frequency difference between high-shore and low-shore anchor sets). *Abbreviations:* ΔAF, absolute allele-frequency difference.

**Supplementary Figure S4.** Robustness of hybrid index estimates to marker set and reference definition. Comparison of strict raw hybrid index (strict HI; fixed ecological references, ΔAF ≥ 0.8; 4 loci) versus semi-diagnostic raw hybrid index from the Q-defined 15/15 reference workflow (ΔAF ≥ 0.5; 29 loci). Each point is one individual; the dashed line indicates 1:1 agreement, and the solid line shows the linear fit. Colours indicate the three ancestry groups (parental *Fucus spiralis*, admixed, parental *F. vesiculosus*)*.*

**Supplementary Figure S5**. Ancestry-depth and heterozygosity relationships using genome-wide ancestry (Q). As Fig. 2A and 2C, but with the genome-wide K = 2 *Fucus* *vesiculosus*-like ancestry proportion (Q; 4,155 LD-pruned SNPs) on the x-axis in place of the 4-locus ancestry index. A. Genome-wide Q versus shore depth (Spearman rho = -0.781, p < 0.001; LOESS + 95% band). B. Genome-wide observed heterozygosity (H_obs; 5,042 SNPs) versus Q (Pearson r = 0.974, 95% CI 0.960-0.983). Points coloured by the three ancestry groups. The relationships match those based on the 4-locus index, confirming they are not artefacts of the small marker panel.

# Supplementary Methods S1. Phlorotannin analysis:

Phlorotannins were quantified from freeze-dried tissue using a Folin-Ciocalteu assay.

**Sample preparation and extraction**

1. Transfer freeze-dried algal tissue to a labelled 1.5 ml microcentrifuge tube.
2. Homogenise the sample in a shaker for 5 min at frequency 30.
3. Weigh approximately 10 mg homogenised material into a new labelled 1.5 ml microcentrifuge tube.
4. Add 1.5 ml 60% acetone.
5. Extract for 1 h on a shaking table at 1500 rpm.
6. Centrifuge for 2 min at maximum speed.
7. Transfer 1 ml of the supernatant to a new labelled 1.5 ml microcentrifuge tube.
8. Evaporate the acetone in vacuo for approximately 1 h, ensuring that samples do not dry out completely. If necessary, add dH2O to prevent complete drying.
9. Dilute the extract to 10 ml with dH2O.
10. Transfer 1.5 ml of the diluted extract to a microcentrifuge tube and centrifuge for 2 min at maximum speed to remove precipitated lipophilic material.

**Folin-Ciocalteu assay**

1. Add 3.5 ml dH2O and 0.5 ml diluted extract to a test tube.
2. Add 0.25 ml Folin-Ciocalteu reagent and mix thoroughly.
3. Add 0.75 ml sodium carbonate solution (Na2CO3, 20 g per 100 ml), mix, and note the time of addition.
4. Prepare a blank using dH2O instead of sample every 20th sample.
5. Incubate samples for 2 h in the dark.
6. Measure absorbance at 725 nm.
7. Calculate phlorotannin concentration using a phloroglucinol standard curve.
